# 

**Published:** 1876-04

**Authors:** 


Vol. VI. April, 1876. No. 4.

### THE SOUTHERN MEDICAL RECORD:

PUBLISHED MONTHLY AT

### ATLANTA, GEORGIA.

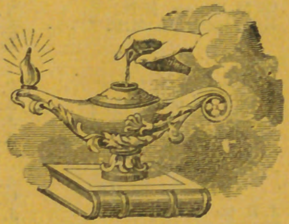

LOCAL EDITORS:

THOS. S. POWELL, M.D.
W. T. GOLDSMITH, M.D.
R. C. WORD, M.D
H. B. LEE, M.D.

ASSOCIATE EDITORS:

T. CURTIS SMITH, M.D............Middleport, Ohio.
SWAN M. BURNETT, M.D .......Knoxville, Tenn.
ALBAN S. PAYNE, M.D...........Markham's, Va.
A. R. KILPATRICK, M.D..........Navasota, Texas.
A. A. LYON, M.D.... ............Shreveport, La.
G. A. BAXTER, M.D ...... .......Chattanooga, Tenn.
J. B. MATTISON, M.D.............Brooklyn, N. Y.

*"QUICQUID PRÆCIPIES ESTO BREVIS."*

ATLANTA, GA.:
JAS. P. HARRISON & CO., PRINTERS AND BINDERS.
1876.

